# A Multiplex Immunosensor for Detecting Perchlorate-Reducing Bacteria for Environmental Monitoring and Planetary Exploration

**DOI:** 10.3389/fmicb.2020.590736

**Published:** 2020-12-16

**Authors:** Ignacio Gallardo-Carreño, Mercedes Moreno-Paz, Jacobo Aguirre, Yolanda Blanco, Eduardo Alonso-Pintado, Isabelle Raymond-Bouchard, Catherine Maggiori, Luis A. Rivas, Anna Engelbrektson, Lyle Whyte, Víctor Parro

**Affiliations:** ^1^Department of Molecular Evolution, Centro de Astrobiología (INTA-CSIC), Madrid, Spain; ^2^Centro Nacional de Biotecnología, CSIC, Madrid, Spain; ^3^Grupo Interdisciplinar de Sistemas Complejos (GISC), Madrid, Spain; ^4^Department of Natural Resource Sciences, McGill University, Sainte-Anne-de-Bellevue, QC, Canada; ^5^Inmunología y Genética Aplicada, S.A. (INGENASA), Madrid, Spain; ^6^Department of Plant & Microbial Biology, University of California, Berkeley, Berkeley, CA, United States

**Keywords:** perchlorate, perchlorate-reducing bacteria, antibody microarrays, biochip, life detection, planetary exploration, Mars, graph theory

## Abstract

Perchlorate anions are produced by chemical industries and are important contaminants in certain natural ecosystems. Perchlorate also occurs in some natural and uncontaminated environments such as the Atacama Desert, the high Arctic or the Antarctic Dry Valleys, and is especially abundant on the surface of Mars. As some bacterial strains are capable of using perchlorate as an electron acceptor under anaerobic conditions, their detection is relevant for environmental monitoring on Earth as well as for the search for life on Mars. We have developed an antibody microarray with 20 polyclonal antibodies to detect perchlorate-reducing bacteria (PRB) strains and two crucial and highly conserved enzymes involved in perchlorate respiration: perchlorate reductase and chlorite dismutase. We determined the cross-reactivity, the working concentration, and the limit of detection of each antibody individually and in a multiplex format by Fluorescent Sandwich Microarray Immunoassay. Although most of them exhibited relatively high sensitivity and specificity, we applied a deconvolution method based on graph theory to discriminate between specific signals and cross-reactions from related microorganisms. We validated the system by analyzing multiple bacterial isolates, crude extracts from contaminated reactors and salt-rich natural samples from the high Arctic. The PRB detecting chip (PRBCHIP) allowed us to detect and classify environmental isolates as well as to detect similar strains by using crude extracts obtained from 0.5 g even from soils with low organic-matter levels (<10^3^ cells/g of soil). Our results demonstrated that PRBCHIP is a valuable tool for sensitive and reliable detection of perchlorate-reducing bacteria for research purposes, environmental monitoring and planetary exploration.

## Introduction

Perchlorate is a stable and soluble toxic anion that is found in the environment from natural and anthropogenic sources (Kounaves et al., 2010). As it accumulates in the food chain (Kirk et al., 2005; Sánchez et al., 2005), it represents a health risk to humans by interfering with thyroid function upon ingestion (Lawrence et al., 2009). On the other hand, natural perchlorate salts have been detected on distinct arid regions as well as on the surface of Mars at relatively high (0.4–0.6 wt%) concentrations (Klein, 1974; Hoffman et al., 2008; Hecht et al., 2009; Glavin et al., 2013; Freissinet et al., 2015). The study of perchlorate metabolism has advanced significantly in a short time, revealing that perchlorate respiration is environmentally ubiquitous and widespread in bacteria and archaea domains (Coates and Achenbach, 2004). Perchlorate-reducing bacteria (PRB) are a phylogenetically diverse group of microorganisms capable of growth by respiring perchlorate as the sole electron acceptor (Youngblut et al., 2016). These dissimilatory perchlorate-reducing bacteria reduce perchlorate to chlorate which is further reduced to chlorite by means of the perchlorate reductase enzyme in the periplasmic compartment. Then, a chlorite dismutase converts chlorite completely to chloride ion with the release of molecular oxygen that can be utilized by a membrane-bound respiratory oxidase (Youngblut et al., 2016). In fact, the removal of perchlorate by means of microbial reduction has been identified as the most efficient method of removing this harmful substance from contaminated environments (Coates and Achenbach, 2004; Hatzinger, 2005; Gu and Coates, 2006; Coates and Jackson, 2009).

Bacterial bioremediation of perchlorate-contaminated water is a viable treatment option, which has spurred both applied (Urbanskya and Schock, 1999; Hatzinger, 2005) and basic (Coates and Achenbach, 2004) science research. As a consequence, PRB have been isolated from a variety of habitats ranging from pristine areas to anthropogenic-contaminated sites (Coates et al., 1999). Phenotypic characterization studies have demonstrated that the known perchlorate-reducing bacteria exhibit a broad range of metabolic capabilities and can thrive in adverse environments such as the Atacama desert, the high Artic and the cold deserts in Antarctica (Wynn-Williams and Edwards, 2000; Parro et al., 2011a; Lay et al., 2013; Youngblut et al., 2016). Bender et al. (2004) developed two degenerate primer sets targeting the chlorite dismutase (*cld*) gene which were subsequently used for the detection of PRB in pristine and diesel-contaminated sediments, and water samples from Antarctica (Youngblut et al., 2016). Other studies monitored a dissimilatory PRB marine microbial community by identifying the different members through 16S rRNA gene sequence analysis (Carlström et al., 2016) and then through metagenomic analysis (Barnum et al., 2018).

Of note, the Phoenix Lander and, more recently, NASA’s Curiosity rover found perchlorates (ClO_4_^–^) in the polar Martian regolith at 0.4–0.6 wt% (Hecht et al., 2009) and within Gale Crater (Freissinet et al., 2015), respectively. These concentrations are considerably higher than those found in terrestrial analogs, such as the Atacama Desert soil surface where it is only 0.03 wt% (Navarro-González et al., 2009) and a maximum of 0.004 wt% in cores and powder samples down to 5 m-depth (Parro et al., 2011a), the soils of the Antarctic Dry Valleys at 10^–5^ wt% (Kounaves et al., 2014) or in the Devon Island ice cap (Nunavut, Canada) at 2 × 10^–7^ wt% (Furdui and Tomassini, 2010). The discovery of perchlorates in the Martian soil has important implications for the detection of organics because the powerful oxidizing properties of perchlorate promote combustion of organics in pyrolytic experiments (Hecht et al., 2009; Carrier and Kounaves, 2015). As such, both the 1976 Viking missions (Klein, 1974) and the Phoenix Lander in 2008 (Hoffman et al., 2008), using gas chromatography-mass spectrometry (GC-MS) instrumentation, failed to detect organics on the surface of Mars, and until very recently, the presence of organic matter on the Red Planet was unclear (Freissinet et al., 2015). Interestingly, even though the presence of perchlorate may constrain the types of detection methods used for identification of organics on Mars, these compounds may also contribute to the creation of appropriate conditions for habitability by lowering the freezing point of water through their low eutectic point and providing a thermodynamically favorable electron acceptor for bacterial respiration (Zorzano et al., 2009; Ojha et al., 2015; Youngblut et al., 2016).

In the past, polyclonal antibodies have been raised against the main enzyme involved in perchlorate reduction metabolism, chlorite dismutase (Cld) (O’Connor and Coates, 2002). This enzyme, highly conserved in PRB, represents an ideal target for the environmental detection of these bacteria. The characterization of these antibodies showed specificity only for cells growing on perchlorate, with little or no cross-reactivity with closely related non-perchlorate-reducing bacteria. Based on this specificity, an Enzyme-Linked ImmunoSorbent Assay (ELISA) was developed for the detection of active perchlorate-reducing bacteria in aqueous samples. Although this immunoprobe has a great potential for monitoring PRB in both mixed cultures and groundwater environmental samples, it fails to analyze solid samples (Coates and Jackson, 2009). Over the last decade, multiplex microarray immunoassay platforms are being used as high-throughput detection methods in complex clinical (Borrebaeck and Wingren, 2009) and environmental samples (Rivas et al., 2008; Parro et al., 2011a; Blanco et al., 2012). Because this technique can be used in the field with different types of samples (solid and liquid) and with little sample handling and preparation, we developed the so-called Life Detector Chip (LDChip) with more than 200 antibodies for detecting microbial molecular markers (Rivas et al., 2008; Blanco et al., 2012). In parallel, we implemented the LDChip into the SOLID (Signs of Life Detector) instrument for remote and automatic analysis (Parro et al., 2011b) for the search for life in future planetary exploration missions (Parro et al., 2005). Both the necessity of environmental monitoring of perchlorate-contaminated areas on Earth and the feasibility of discovering PRB on Mars led us to develop a multiplex immunoassay for detecting bacteria capable of canonical perchlorate respiration.

Herein, we report the characterization of a set of 24 antibodies targeting whole bacterial strains, exopolysaccharides, and key enzymes involved in perchlorate metabolism, as well as the development and validation of an antibody microarray based on antibody reactivity and specificity to monitor the environmentally dominant PRB species and some metabolic indicators in natural samples. Although several immunological methods have been applied during the last decades to develop tools for the detection of biomarkers in natural samples, to our knowledge, this is the first time that an immunosensor based on microarray technology has been developed to detect PRB in a multiplex assay.

## Materials and Methods

### Immunogens and Production, Purification and Labeling of Antibodies

Strains and culture conditions of PRB used as a source of immunogens are shown in Table 1, as well as the antibodies produced and used in this study. Bacteria were grown to 10^8^ cells mL^–1^ under anaerobic conditions either in medium A (basal-phosphate buffered medium, acetate carbon source, chlorate electron acceptor), medium B (PIPES buffered medium, acetate carbon source, chlorate electron acceptor, 2% NaCl), or medium C (bicarbonate buffered medium, acetate carbon source, perchlorate electron acceptor, 3% NaCl) (Bender et al., 2002), and monitored by optical absorbance at 600 nm with an 840-208100 Genesys 10S UV/VIS (Thermo Scientific) spectrophotometer. Cells were collected by centrifugation and resuspended in one-tenth of the culture volume of 10% sterile trehalose dehydrate and lyophilized. To prepare the exopolysaccharide (EPS) fraction, 12 mL of culture medium were filtered through 0.22 μm pore size filter and 30 mL of isopropanol were mixed with the cell-free medium and incubated at −20°C for 12 h for EPS precipitation. The precipitated was collected by centrifugation at 6,500 *g* for 16 min at 4°C. The supernatant was discharged and the resulting pellet suspended in 0.4 mL phosphate buffer (pH = 7.2) and transferred to a 2 mL tube. One mL of isopropanol was added to re-precipitate and concentrate the EPS fraction. Tubes were stored at −20°C for 10 days. The EPS collected by centrifugation at 6,000 *g* for 17 min at 4°C and then dried under a flow of nitrogen.

**TABLE 1:** List of the strains and immunogens used to produce the antibodies.

Ab code	Bacterial strain as immunogen	Class of Proteobacteria	Medium/days	LOD (Cells/mL)	LOD TOP-PRB (Cells/mL)
L1C1	*Azospira suillum* PS	Beta-	A/21	<10^3^	<10^3^
L2C1	*Magnetospirillum bellicus* VDY	Alpha-	A/21	<10^2^	<10^2^
L3C1	*Ideonella dechloratans*	Beta-	A/21	<10^3^	<10^3^
L4C1	*Dechlorobacter hydrogenophilus* LT-1	Beta-	A/4	<10^3^	<10^4^
L5C1	*Propionibrio militaris* MP	Beta-	A/21	<10^3^	<10^3^
L6C1	*Dechloromonas agitata* CKB	Beta-	A/5	<10^3^	<10
L7C1	*Magnetospirillum* sp. WD	Alpha-	A/4	<10^5^	<10^5^
L8C1	*Azospira* sp. ZAP	Beta-	A/21	<10^3^	<10^3^
L9C1	*Shewanella algae*	Gamma-	B/21	<10^2^	<10^2^
L10C1	*Dechloromarinus chlorophilus* NSS	Gamma-	B/21	<10^4^	<10^5^
L11C1	*Dechloromonas aromatica RCB*	Beta-	A/4	<10^3^	<10^3^
L12C1	*Arcobacter* sp. *CAB*	Epsilon-	C/78	<10^3^	<10^5^
L1S2	*Azospira suillum* PS EPS	Beta-	A/21	<10^4^	<10^5^
L2S2	*Magnetospirillum bellicus* VDY EPS	Alpha-	A/21	–	–
L3S2	*Ideonella dechloratans* EPS	Beta-	A/21	–	–
L4S2	*Dechlorobacter hydrogenophilus* LT-1 EPS	Beta-	A/4	<10^3^	<10^4^
L6S2	*Dechloromonas agitata* CKB EPS	Beta-	A/5	<10^2^	<10
L8S2	*Azospira* sp. ZAP EPS	Beta-	A/21	<10^3^	<10^3^
L11S2	*Dechloromonas aromatica RCB* EPS	Beta-	A/4	<10^5^	<10^5^
L12S2	*Arcobacter* sp. *CAB* EPS	Epsilon-	C/78	<10^6^	–

*The performance of the antibodies is indicated as the limit of detection (LOD) expressed as number of cells per mL of suspension. All strains are perchlorate reducers, except for the strains Ideonella dechloratans and Shewanella algae, which are chlorate reducers. All strains were cultivated in anaerobiosis in medium A, B, and C as indicated in Section “Materials and Methods.” Antibody code: L1-12, different antibody; C1, whole cell extract as immunogen; S2, extracellular EPS fraction as immunogen*.

The immunogens were prepared by using 10^9^ cells mL^–1^ (C1 in the antibody code in Table 1) or 0.7 mg dried weight of bacterial exopolysaccharides (S2 in the antibody code). Polyclonal antibodies were produced by immunizing rabbits as reported previously in Parro et al. (2005). All of the antibodies, as well as the pre-immune sera, were purified by protein A affinity column (Sigma-Aldrich, PURE1A) to obtain the IgG fraction and then fluorescently labeled with Alexa 647 fluorochrome (Molecular Probes, OR, United States) as described in Rivas et al. (2008).

### Antibody Microarray Production

Protein A-purified antibodies (IgG fraction) and their corresponding pre-immune sera were printed by triplicate spot pattern on epoxy-activated glass slides (Arrayit, CA, United States) at a concentration of 0.8 mg mL^–1^ in protein printing buffer 1× (Whatman International, United Kingdom) plus 0.01% Tween 20, using a MicroGrid II TAS arrayer (Biorobotics, Genomic Solutions, United Kingdom) as previously reported (Parro et al., 2011a). The resulting antibody microarray, from now on PRBCHIP, was printed in a 3 × 8 identical replicate format to fit into a 3 × 8 wells gasket for 24 simultaneous analyses. A serial dilution of a fluorescent frame using a fluorescently labeled pre-immune antibody was printed for checking microarray quality and the easy localization of the microarray pattern. Redundant spots that contained BSA, protein printing buffer or pre-immune antiserum were used as blank control spots.

### Fluorescence Sandwich Microarray Immunoassay (FSMI)

One milliliter of each PRB culture, as well as natural samples obtained from a variety of ecosystems, were ultrasonicated for 5 cycles of 30 s with 30 s pause on ice using a manual sonicator (Dr. Hielscher 50 W DRH-UP50H sonicator, Hielscher Ultrasonics, Berlin, Germany) for sample homogenization and partial cell lysis, and immediately used as test sample for the immunoassay. For FSMI, the whole slides were blocked with Bovine Serum Albumin (BSA) as described previously (Rivas et al., 2008; Parro et al., 2011a). The FSMIs were performed in the laboratory as follows: after blocking, different quantities of each immunogen were mixed with TBSTRR buffer (0.4 M Tris-ClH, pH 8, 0.3 M NaCl, 0.1% Tween 20) and 50 μL aliquots were incubated with the PRBCHIP for 1 h at room temperature. After incubation and washing with the same buffer, 50 μL of the corresponding fluorescent antibody or a fluorescent mixture composed of the antibodies raised against all strains plus A-PCR and A-Cld antibodies (TOP-PRB mix) was added for an additional incubation of 1 h at 4°C, washed again, scanned for fluorescence at 635 nm excitation peak, and the images quantified and analyzed as described in Blanco et al. (2014).

### Antibody Graph Associated With the Antibody Microarray and Deconvolution Analysis of Experimental Data

An antibody graph describes an antibody microarray for sandwich immunoassays and its antibody cross-reactivity events as a complex network (Rivas et al., 2008). The antibody graph G has *N* nodes (number of antibodies represented in the microarray) and *l* links (number of cross-reactions) and has an associated matrix *G* of size *N × N.* The methodology to calculate the antibody graph associated with the PRBCHIP from the cross-reactivity matrix *G* obtained after testing one by one all the pairs antibody-immunogen is described in Rivas et al. (2011).

The information embedded in the antibody graph allows the application of a deconvolution algorithm to disentangle correlations and distinguish the true reactions from the ones coming from different levels of cross-reaction. Although the reader should refer to Rivas et al. (2011) and Blanco et al. (2014, 2015a,b) for details, in summary, the method is based on the following statement: the fluorescence intensity of one antibody spot on the microarray can be approximated as the sum of the contributions of all the antibodies that cross-react with it. In practice, this method yields a deconvoluted signal associated with each fluorescence signal and analyzing these two sets of values we can obtain valuable information about the existence or not of the different organisms composing the sample. In particular, it gives rise to three different possibilities for each antibody printed on the microarray: (i) type I antibody, its cognate antigen is not present in the sample under study; (ii) type II antibody, its cognate antigen is not present in the sample, but a related antigen is (in case II.a the related antibody can be or not in the microarray, while in case II.b the related antibody is certainly not in the microarray, and therefore is unknown); and (iii) type III antibody, either its cognate antigen or a closely related antigen is present in the sample. Furthermore, A-type antibodies have forward cross-reactions (i.e., out-going arrows from their corresponding node in the antibody graph), and B-type antibodies do not.

Finally, note that the antibody graph and the deconvolution method associated with it do not only disentangle the cross-reactivities due to the polyclonal nature of the antibodies, but they also take advantage of them. The main benefits are twofold: first, our technique can unmask false positives as it warns us when the cross-reactivities associated are not the expected ones; second, the deconvolution method allows us to detect signals whose related antibody can be or not in the printed microarray, making the PRBCHIP capable of detecting a large number of biomarkers beyond those present in the microarray.

### Validating of the PRBCHIP With Enriched Cultures and Environmental Samples

The PRBCHIP was tested and validated by analyzing its immunodetection capability with 66 environmental bacterial isolates and 14 (named A to N in Table 3 and Supplementary Table 2). PRB-enriched cultures from industrial bioreactors (American Pacific^TM^) treating perchlorate-contaminated groundwaters. In addition, samples from these bioreactors were taken at different depths and over several days to identify the microbial agents responsible for the contamination. Aliquots of water were taken with a sterile syringe and filtered through a 0,22-μm pore size nitrocellulose filter (Merck Millipore). The biomass collected in each filter was suspended in 1 mL of TBSTRR buffer by scraping it with a spatula into a 15 mL tube. Then, cells were disrupted by ultrasonication as described above, and 50 μl of the resulting cell extract was used for analysis by FSMI. The detection of positive signals obtained with the PRBCHIP at different depths was additionally confirmed by cloning and sequencing the 16S rRNA gene, and with the corresponding analyses of the operational taxonomic units (OTUs) obtained (Puente-Sánchez et al., 2016). Additionally, natural samples collected during a 2013 summer campaing to the High Canadian Artic at the Mars Station (McGill University, Canada) in a water stream next to a big dome from Lost Hammer (LH) perennial spring (79°4.608 N; 90°2.739 W) were also analyzed by FSMIs. Samples from a salt crust (LH-SC) and a sediment (LH-Sed) 5 cm underneath the salt crust were collected by using a sterile spatula and stored in sterile tubes or plastic bags. Up to 0.5 g of samples were subjected to an aqueous extraction in TBSTRR buffer either by sonication as described above and then filtered through 20-μm nitrocellulose filters or let to sediment for 5 min to remove the coarse material. Both filtrates and supernatants were considered as crude environmental extracts and used directly for FSMI with PRBCHIP.

To discard potential false-positive signals that might arise from the binding of mineral particles to antibodies, samples were subjected to a heat treatment (500°C, 3 h) to denature or disintegrate the organic molecules. They were then processed as the non-treated samples. Signal detection after this treatment will correspond to the binding of mineral particles while positive immunodetections will disappear in the heated sample compared with the non-heated one. Besides, every FSMI had a negative control (incubation with the buffer only) that was developed with the same fluorescent antibody mixture. The measured signals were subtracted from each assay within the chip to eliminate the effect of non-specific fluorescence.

### Dot Blot Assays

On a nitrocellulose membrane piece, we applied triplicate spots of preparations of either perchlorate reductase or chlorite dismutase at 1 mg mL^–1^ concentration each, both obtained from *Dechloromonas agitata* strain CKB as previously described (Kengen et al., 1999; O’Connor and Coates, 2002). Bovine Serum Albumin (BSA) was used as a negative control. The membranes were blocked with BSA and incubated with different antibodies: A-PCR A/B, A-Cld, A-295, and A-PCR, at their corresponding working dilution in TBSTRR with 0.1% BSA. After washing, they were incubated with an HRP-conjugated anti-rabbit IgG secondary antibody and then revealed with Luminol (Sigma–Aldrich #*123072*) and hydrogen peroxide following supplier instructions. Finally, the membranes were observed with a *trans*-illuminator coupled to a photo camera.

### DNA Extraction and Sequencing From PRB Cultures and Natural Samples

The PowerLyzer Ultra Clean Microbial DNA isolation kit (Mo Bio Laboratories Inc., CA, United States) was used to extract total DNA from 1 mL of the bacterial cultures or 0.5 g from LH samples and sludge samples collected from industrial bioreactors (BR) treating perchlorate contaminated groundwaters. The V3–V5 region of the 16S rRNA gene was amplified using key-tagged eubacterial primers based on design by Sims et al. (2012) for bacterial cultures and BR samples and based on Chih-Ying et al. (2012) for LH DNA. The 16S rRNA gene amplicons were sequenced by Roche 454-GS-FLX+-Titanium pyrosequencing system (Sequencing service, UC Berkeley, California). DNA sequences were deposited on NCBI Genebank under accession numbers detailed in Supplementary Table 1. Additionally, different isolates were obtained from high Arctic LH samples, although there were no perchlorate reducers among them. To confirm the presence of a *cld* gene, a PCR amplification was carried out using *cld* specific primers (Supplementary Table 2), and the amplicon was sequenced and compared against the NCBI database for sequence similarities.

### Culture Enrichment and Immunostaining of Bacterial Cells With Anti-Cld Antibody

A modified immunofluorescence microscopy procedure described by Pirbadian et al. (2014) was performed to test the presence of the periplasmic protein Cld in LH samples and enrichment cultures. Briefly, 20 g of salt crust LH samples were diluted in sterile distilled water (1:1 w/v) and, after letting coarse material and minerals sediment, 500 μL of the liquid were inoculated into bottles with 100 mL of medium for chlorate-respiring bacteria (DSMZ 908) under anaerobic conditions. Bottles were incubated at 4°C to stimulate psychrophilic growth. Some turbidity was observed after 4 months of incubation, and 1 mL of culture was fixed with 37% formaldehyde (200 μL) in PBS (800 μL) for 1 h at room temperature (RT), filtered through 0.22 μm polycarbonate filters (ClearLine), and rinsed with 10 mL of PBS. Filters were then transferred to a permeabilizer solution of 0.1% Triton X-100 in PBS for 45 min at RT and washed three times in PBS. They were soaked in blocking solution (1× PBS and 1% BSA) for 5 min at RT to block non-specific binding sites and then incubated at RT for 1 h.

After blocking non-specific binding with a solution of 1% BSA in 1X PBS for 5 min at RT, the filter was incubated with the diluted (1/50) anti-Cld rabbit polyclonal antibody (O’Connor and Coates, 2002) at RT for 1 h. The filter was rinsed four times in PBS and finally incubated with a commercial goat anti-rabbit IgG Alexa 555-conjugated antibody (Molecular Probes) at RT. Then filters were rinsed twice in PBS and stained with DAPI (Biotium) for 5 min at RT in darkness. After rinsing twice in PBS, they were mounted in Vectashield antifade medium (Vector Laboratories) and observed under an Axioskop 2 microscope (Zeiss). Immunofluorescence images were taken with an AxioCam MRc 5camera (Zeiss). Cultures of *Escherichia coli* strain HFR and *Dechloromonas agitata* strain CKB were used as negative and positive controls for Cld, respectively. Additional immunofluorescence microscopy assays were directly performed with the LH salt crust suspension to assess the presence of perchlorate-reducing bacteria or chlorite-detoxifying activities. A volume of 7.5 mL (1:1 w/v) of a fresh sample was fixed with 37% formaldehyde (1.5 mL) in PBS (6 mL) for 1 h at room temperature (RT). Samples were then filtered and immunostained with anti-Cld as above.

### Molecular Phylogenetic Analysis of the Enrichment Culture

Total DNA was extracted from 2 mL of enrichment cultures obtained from LH samples after centrifugation at 10,000 *g* for 10 min. Cells were suspended into 100 μL of sterile distilled water and then lysed by thermic shock at 94°C during 5 min. Aliquots of 10 μL were used for bacterial 16S rRNA gene PCR amplification using universal pair primers 16SF (forward, 5′-AGAGTTTGATCCTGGCTCAG-3′) and 1492R (5′-GGTTACCTTGTTACGACT-3′). PCR conditions were as follows: 3 min at 94°C, followed by 35 cycles (denaturation for 30 s at 94°C, annealing for 30 s at 94°C and elongation for 1 min at 94°C) and a final elongation step for 4 min at 94°C. PCR products were checked by agarose gel electrophoresis (Amersham Biosciences) and were purified using the QIAquick PCR Purification Kit (QIAGEN). The amplicons were cloned using the TOPO TA Cloning Kit (K4575, Invitrogen). Ligation was performed using 4 μL of the purified PCR products into a final volume of 6 μL with 1 μL of pCRII-TOPO vector (Invitrogen) at 4°C overnight. 2 μL of each ligation were added into a tube of competent cells TOP10 (Invitrogen), using 1 μL of pUC19 vector (Invitrogen) as positive control. Cells were transformed following manufacturer’s instructions. Up to 96 colonies were cultivated and used to extract plasmid DNA with an EpMotion 5075Vac fluid management robot (Eppendorf) and checked by agarose gel electrophoresis. Sequencing reactions were done with the Big Dye Terminator Kit 3.1 (Applied Biosystems) and sequenced in a Seqstudio DNA Analyzer (Applied Biosystems) using the M13-forward, M13-reverse and 926R [5′-CCGTCAATTC(A/C)TTTGAGTTT-3′primers] (Watanabe et al., 2001). Sequences were assembled with the package SeqMan Pro sequence assembler package (DNASTAR, Lasergene Molecular biology). Taxonomical information and identification were carried out by comparing the consensus sequences obtained against the NCBI NR database with BLAST (Altschul et al., 1997).

## Results

### An Antibody Microarray for Multiplex Detection of Perchlorate-Reducing Bacteria

**FIGURE 1:**
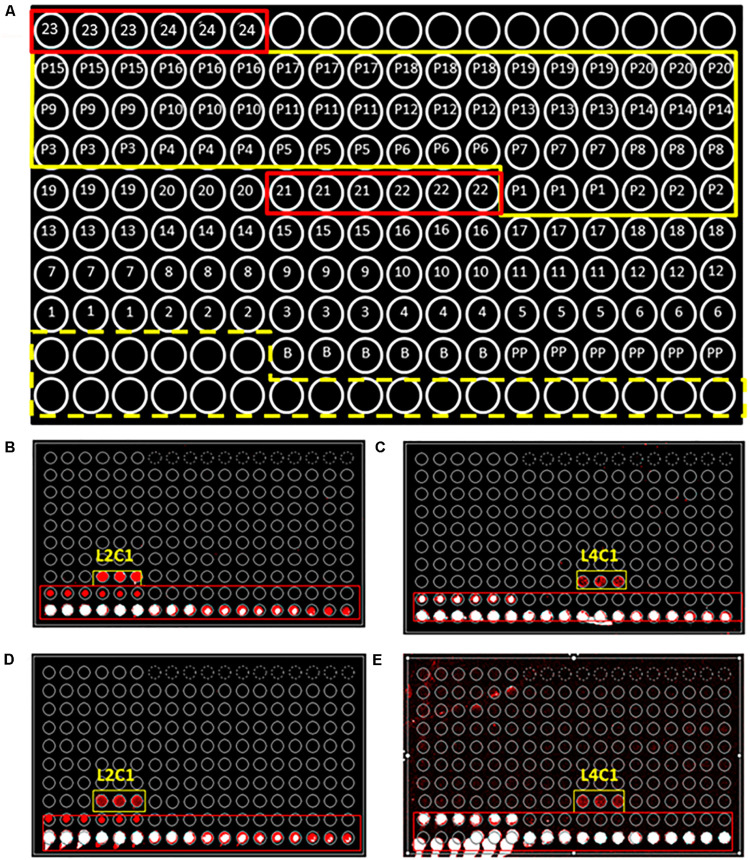
PRBCHIP, an antibody microarray for detecting perchlorate reducing bacteria. **(A)** Schematic of the antibody printing pattern layout (by triplicate) in the PRBCHIP as indicated in Table 1 and in Section “Materials and Methods.” Empty circles correspond to a serial dilution of a fluorescent antibody printed as a control for fluorescence (marked with a yellow dashed line); circles 1–20 represent antibodies raised against different strains; circles 21–24 indicate antibodies produced against proteins (red rectangles); P1–P20 indicate their corresponding pre-immune antibodies (marked with a continuous yellow line); circles labeled as B (BSA) and PP (Protein Printing Buffer) were used as negative control spots. **(B,C)** Images obtained after fluorescence sandwich microarray immunoassay (FSMI) with PRBCHIP by using cell lysates of *Magnetospirillum bellicus* VDY **(B)** and *Dechlorobacter hydrogenophilus LT-1* **(C)** strains as sample. Immunoassays were revealed with anti-*M. bellicus* VDY (L2C1) and anti-*D. hydrogenophilus LT-*1 (L4C1) antibodies, respectively. **(D,E)** Images obtained for the same samples and revealed with TOP-PRB fluorescent mix, made up of all anti-PRB antibodies shown in Table 1 plus A-Cld and A-PCR antibodies. Red and white spots are fluorescence signals corresponding to positive immunodetections.

We produced a collection of 20 polyclonal antibodies against diverse perchlorate-reducing bacterial strains and 4 antibodies against two of the key proteins involved in perchlorate reduction (see Table 1 and section “Materials and Methods”). Antibody microarrays containing the 24 different antibodies were produced and used to titrate and characterize the antibodies (Figure 1). The antibodies were labeled (hereafter tracer antibodies) and titrated by FSMI by using a constant immunogen concentration and serial dilutions of the antibody. The corresponding microarray images were quantified and, from the calibration curves obtained (data not shown), we inferred that the optimal antibody concentration (IgG fraction) was between 1 and 3 μg mL^–1^ for most of the antibodies produced (Table 1).

**FIGURE 2:**
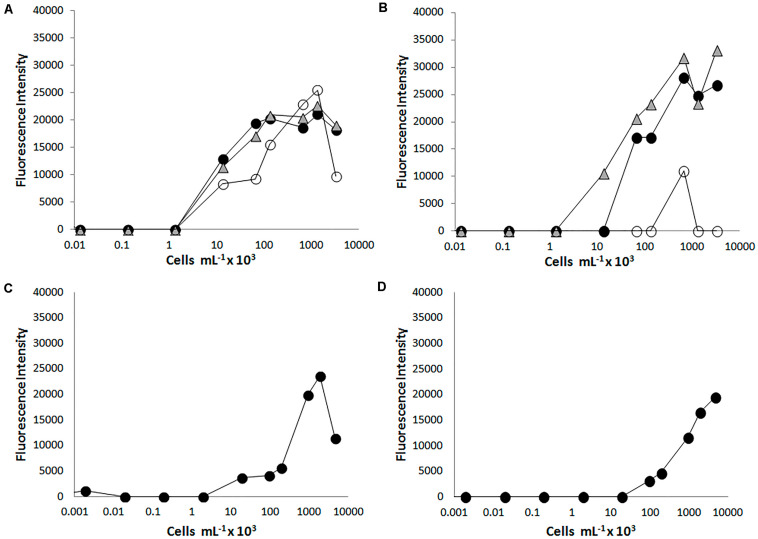
Testing the PRBCHIP sensitivity by fluorescent sandwich microarray immunoassay (FSMI). We assayed serial dilutions of cell cultures as samples for FSMI using their corresponding fluorescent antibodies or the TOP-PRB mix. The fluorescent signals in the microarray were quantified and plotted against the sample concentration. **(A)** Example of the calibration curve for antibody L1C1 raised against *Azospira suillum* PS and revealed with its tracer antibody and **(B)** with the TOP-PRB mix, respectively. When *Azospira suillum* PS was used as tested sample, besides L1C1 (black circles) and L1S2 (white circles), also L8C1 (triangles) showed a comparable signal at high sample concentrations. **(C)** Calibration curve for the antibody L4C1 raised against *D. hydrogenophilus LT-1* revealed with its tracer antibody and **(D)** revealed with the TOP-PRB mix. In this case, only L4C1 showed positive immunodetections.

The sensitivity of each immunogen-antibody pair was determined by FSMI using serial dilutions of whole-cell lysates, EPS fractions, or each purified protein with their corresponding optimal antibody concentrations. The microarray images obtained with different immunogen dilutions were quantified and calibration curves were produced (Figure 2) to determine the limit of detection (LOD) for each antibody (Table 1). The sensitivity ranged from <10^3^ cells mL^–1^ for anti-*Magnetospirillum bellicus* (L2C1) and anti-*Shewanella algae* (L9C1) to 10^5^ cells mL^–1^ for anti-*Magnetospirillum* sp. (L7C1) antibodies. Most of the antibodies showed a mean LOD of 10^3^–10^4^ cells mL^–1^, while the antibodies produced against the EPS fraction of *Desulfobacter hydrogenophilus* (L4S2), *Azospira* sp. (L8S2), *Dechloromonas aromatica* (L11S2), and *Arcobacter* sp. (L12S2) rendered the highest LODs with their immunogen in a sandwich assay. In addition, no signal detections were observed for the antibodies anti-*Magnetospirillum bellicus* EPS (L2S2) and anti-*Ideonella dechloratans* EPS (L3S2).

**FIGURE 3:**
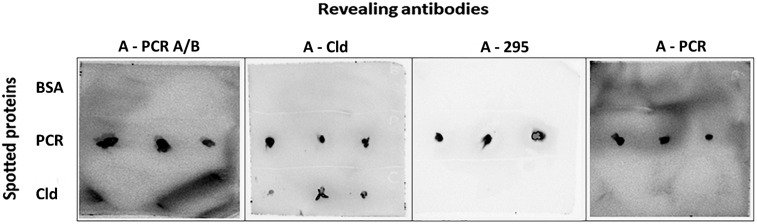
Immunoblot (dot blot) assay for detecting proteins involved in perchlorate metabolism. Perchlorate reductase (PCR) and chlorite dismutase (Cld) proteins were spotted onto nitrocellulose membranes (1 mg mL^–1^), tested with A-PCR_A/B, A-Cld, A-295, and A-PCR primary antibodies and revealed with the HRP-conjugated anti-rabbit IgG secondary antibody and Luminol. Positive signals were detected for PCR with all tested antibodies, while Cld was only detected by its antibody (anti-Cld). BSA was used as negative control.

Similarly, the antibodies produced against proteins showed a poor performance by FSMI analysis using purified perchlorate reductase and chlorite dismutase from *D. agitata* CKB as test samples. Only dot blot tests on nitrocellulose membranes showed positive immunodetections and specific signals with anti-perchlorate reductase antibodies (A-PCR A/B, A-295, and A-PCR), while anti-chlorite dismutase (A-Cld) antibody recognized both the perchlorate reductase and the chlorite dismutase (Figure 3). However, these antibodies recognized these proteins as part of the crude cell extracts from *D. agitata* CKB and *Dethiobacter alkaliphilus strains* assayed in PRBCHIP (Table 2).

**TABLE 2:** Validating PRBCHIP with several perchlorate reducers and other cultures in different conditions (aerobic or anaerobic).

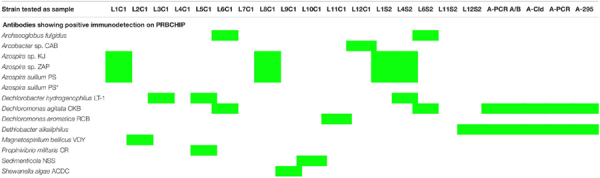

Positive immunodetections (shadowed boxes) on the antibodies printed on the PRBCHIP (columns) when different bacterial strains or fractions are assayed by sandwich immunoassay revealed with a mixture containing all fluorescent-labeled tracer antibodies (TOP-PRB mix). * Grown under aerobic conditions. Strains tested in blind sandwich immunoassay with PRBCHIP*: Acidovorax ebreus TPSY, Archaeoglobus fulgidus, Arcobacter sp. CAB, Azoarcus sp., Azoarcus toluvorans, Azospira oryzae, Azospira sp. KJ, Azospira sp. ZAP, Azospira suillum PS (aerobic and anaerobic), Bacillus cereus, Bacillus mojavensis, Dechlorobacter hydrogenophilus LT-1, Dechloromonas agitata CKB, Dechloromonas aromatica RCB, Dechloromonas hortensis DMSZ 15637, Dechloromonas sp. JJ, Dechlorospirillum sp. WD, Dechlorospirillum sp. DB, Dechlorospirillum sp. M1, Denitromonas SFB1, Denitromonas SFB2, Denitromonas SFB3, Desulfotomaculum acetoxidans, Desulfuromonas palmitatis, Dethiobacter alkaliphilus, Escherichia coli^$^, Geobacter bemidjiensis, Geobacter chapellei, Geobacter gobicium, Geobacter hydrogenophilus, Geothrix fermentans, Holophaga foetida, Magnetospirillum bellicus VDY, Marinobacter VCB, M. triosporum, Propinivibrio militaris CR, Pseudogulbenkiania ferrooxidans COSMO, Pseudomonas sp. PK, Pseudomonas stutzeri^$^, Pyrobaculum islandicum, Sedimenticola sp. NSS (aerobic and anaerobic), Sedimenticola selenatireducens, Shewanella algae ACDC, Thauera aromatica, and Thermincola ferriacetica. ^$^*These organisms are incapable of reductive perchlorate respiration.

**TABLE 3:** Antibodies in PRBCHIP showing positive immunodetections with samples from enriched cultures obtained from contaminated industrial reactors.

	Revealing fluorescent antibodies		
Sample	TOP-PRB mix	L6C1	L6S2
A	L6C1	L6C1	L6C1, L6S2
B	L6C1, L6S2	L6C1, L6S2	L6C1, L6S2
C	L6C1	L6C1, L6S2	L6C1, L6S2
D	L6C1	L6C1, L6S2	L6C1, L6S2
E	L6C1, L6S2	L6C1, L6S2	L6C1, L6S2
F	L6C1, L6S2	L6C1	L6C1, L6S2
G	L6C1, L6S2	–	–
H	L6C1	L6C1	L6C1
I	L6C1, L6S2	L6C1	L6C1
J	L6C1, L6S2	L6C1	L6C1
K	L6C1, L6S2	L6C1	L6C1
L	L6C1, L6S2	L6C1	L6C1
M	L6C1, L6S2	L6C1	L6C1
N	L6C1	–	–

*The sandwich immunoassay was revealed with TOP-PRB mix, L6C1, and L6C2 as indicated in Section “Materials and Methods.” L6C1 and L6S2, antibodies raised against Dechloromonas agitata CKB cells (C1) and EPS (S2). A to N, American Pacific reactor samples (see section “Materials and Methods”)*.

In order to develop a multiplex assay, we tested the performance and the sensitivity of each antibody when they were forming part of a multiplex tracer mixture (TOP-PRB mix) of all the fluorescent tracer antibodies at their working dilution, and using each cell lysate as a test sample. Again, we determined the LOD from the calibration curves for each pair cell-lysate/antibody (Figure 2 and Table 1). The LOD of all the antibodies was similar to that obtained using the single tracer antibody or increases in some of the cases, except for *D. agitata* antibodies (L6C1 and L6S2), where the sensitivity dropped nearly one order of magnitude.

**FIGURE 4:**
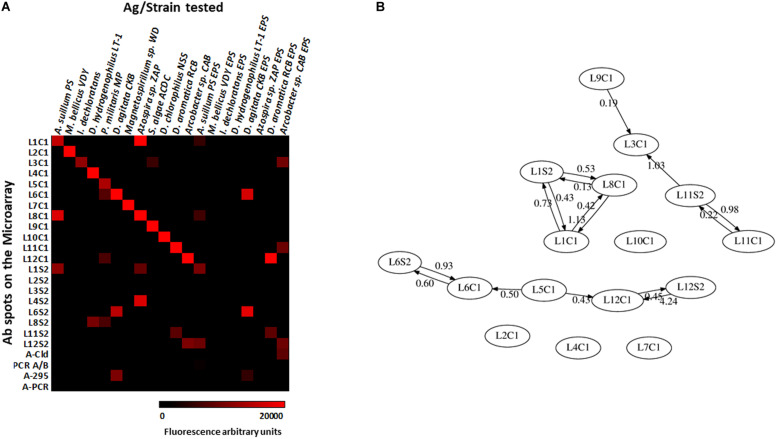
Testing the specificity of the antibodies with the PRBCHIP. Immunogens corresponding to whole-cell extracts of each perchlorate reducing strain, its EPS fractions and proteins were tested by FSMI with its corresponding fluorescent antibody at its appropriate working dilution (see section “Materials and Methods”). **(A)** Specificities and cross-reactions between different antibodies and immunogens. Microarray images were scanned for fluorescence, quantified and plotted in a heatmap. Each column corresponds to a single PRBCHIP assay with the corresponding strain (Table 1) or protein tested (see section “Materials and Methods”) as the immunogen, while each row represents the antibody code for each printed antibody (as in Table 1) on the microarray. **(B)** Antibody graph G of 16 nodes (antibodies with low performance were not considered for this analysis) and 28 links associated with our antibody microarray. Each node represents one antibody. The link (arrow) from antibody *j* to antibody *i* represents cross-reactivity of weight *G*_ij_, where *G*_ij_ is the extent of cross-reactivity between antibodies *i* and *j* referred to the cognate immunogen of antibody *j*. Self-loops (*G*_jj_ = 1) are not shown for clarity.

We evaluated the PRBCHIP as a multiplex detection system by testing the specificity and cross-reactivity of each antibody one by one using their cognate immunogen as a test sample and the corresponding fluorescently labeled antibody as a tracer by FSMI (Figure 1). Fluorescence intensity (*F*) of each antigen-antibody pair was calculated as described in Rivas et al. (2011) and only positive signals were considered by FSMI when the fluorescent signal intensities for each antibody exceeded 2.5 times the background level. After image analysis, fluorescence quantification and data arrangement into a heat map plot (Figure 4A), we applied the deconvolution method to develop the antibody graph associated with the PRBCHIP (Figure 4B; see section “Materials and Methods”). The antibody graph shows a map of the interactions between different antigen/antibody pairs and quantifies the strength of such cross-reactions. This one contains 16 nodes (antibodies) instead of 24 because we excluded the antibodies raised against the EPS fraction for *M. bellicus* VDY, *I. dechloratans*, *D. hydrogenophilus* LT-1 and *Azospira* sp. ZAP and the proteins PCR, PCR A/B, Cld and 295, due to their poor performance parameters by FSMI. The graph indicates that in most cases the antibodies were quite specific for their corresponding antigenic strain or for other taxonomically related strains. Specificity was observed in the case of antibodies raised against *Magnetospirillum* (L2C1; L7C1), *I. dechloratans* (L3C1), *Dechlorobacter hydrogenophilus* LT-1 (L4C1) and *Dechloromarinus chlorophilus* NSS (L10C1) strains which reacted exclusively with their corresponding antigen (type B antibodies, see section “Materials and Methods”).

Some consistent and reproducible cross-reactions were detected in PRBCHIP (Figure 4A): antibodies produced against cell extracts, such as anti-*Azospira suillum* PS (L1C1), *Arcobacter* sp. CAB (L12C1) and the antibodies produced against *Dechloromonas* strains (L6C1; L11C1) reacted strongly with their corresponding EPS-fractions. Conversely, the antibodies L2S2, L3S2, L4S2, and L8S2 did not give positive signals against their own antigens and therefore are not present in the graph.

Finally, only a few cross-reactions were found between apparently not related genera. For instance, *anti-Propinivibrio militaris* MP (L5C1) cross-reacted with anti-*D. agitata* CKB (L6C1), both members of the Rhodocyclales family, and also with the antibody produced against the *Epsilonproteobacteria Arcobacter* sp. CAB (L12C1). Similarly, the anti-*S. algae* (L9C1) cross-reacted very weakly with anti-*I. dechloratans* (L3C1) cell extract.

### Validation of PRBCHIP and the Deconvolution Method With Environmental Isolates

Whole-cell lysates of 66 disparate cultures of chlorate and perchlorate-reducing bacteria (Coates lab collection, UC Berkeley) were used as samples in a blind test to assess the PRBCHIP. Only 13 out of the 66 strains tested showed positive immunodetections with some of the antibodies on the PRBCHIP (Table 2), suggesting that the antibodies are highly specific, in agreement with the cross-reactivity analysis shown above (Figure 4). Even culture conditions may affect the immunodetection, as it was the case for a cell extract from *A. suillum* grown under aerobic conditions, which did not show any positive immunodetection (Table 2), indicating that the true antigens recognized by the antibodies were only expressed under anaerobic conditions. This has been previously described for cultures of *D. agitata* CKB strain growing under anaerobic conditions in the presence of perchlorate as electron acceptor (O’Connor and Coates, 2002).

To validate the PRBCHIP as a useful tool for detecting PRB in complex samples that might have the capacity of dissimilatory perchlorate reduction, we analyzed a set of samples from contaminated soil reactors (American Pacific^TM^) by FSMI (Table 3). The results showed strong immunodetection signals with anti-*D. agitata* CKB antibodies both from cellular and EPS fractions (L6C1 and L6S2 antibodies, respectively), using the TOP-PRB mix or the individual tracer antibodies (not shown). No signals were detected in the spots corresponding to pre-immune antiserum or other antibodies, indicating that either the samples were enriched in *D. agitata* related strains, or the PRBCHIP does not contain the antibodies for other PRB potentially present in the samples.

**FIGURE 5:**
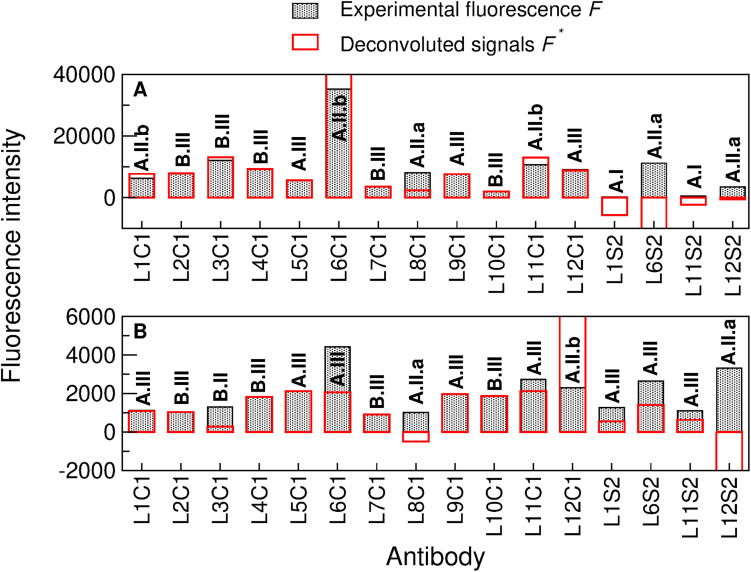
Deconvolution method applied to two complex natural samples by sandwich microarray immunoassay (FSMI). **(A)** Deconvolution of the American Pacific sample 140-10_07/07 and **(B)** deconvolution of the Lost Hammer sediment sample (LH-Sed) after performing both immunoassays with the mixture of all fluorescent-labeled tracer antibodies (TOP-PRB mix). Filled black bars represent the experimental fluorescence intensities *F* and red bars represent the deconvoluted signals *F*′ [see ^(40–41)^ for details on obtaining *F*′ from *F* and the matrix *G* associated to the antibody graph G]. Note that the experimental fluorescence intensities are ≥0 (as they were directly obtained from the FSMI), while the deconvoluted signals might be positive, zero or negative. Analyzing whether the experimental signal of each antibody is positive or close to zero and whether its deconvoluted signal is positive, zero or negative, we obtain a code for each organism (in bold) that yields reliable information about its existence or not in the sample [see supporting information of Blanco et al. (2015b)].

The deconvolution method presented in Rivas et al. (2008) and thoroughly explained in the Supporting Information of Blanco et al. (2015a) was applied to elucidate whether each experimentally recorded fluorescent signal was due to its cognate immunogen or to a taxonomically closely related one (see section “Materials and Methods” for details). The deconvolution results applied to sample 140-15_07/07 (Figure 5A) indicated that anti-*A. suillum* PS EPS (L1S2) and anti-*D. aromatica* RCB EPS (L11S2) were type I antibodies, revealing that the corresponding microorganisms or immunogens were not in the sample; anti-*Azospira* sp. ZAP (L8C1), anti-*D. agitata* CKB EPS (L6S2) and *Arcobacter* sp. CAB EPS (L12S2) were type A.II.a antibodies, so they were not in the sample, and their brightness in the fluorescence image was due to the presence of a nearby microorganism or molecule that can be represented in the graph or not. Anti-*A. suillum* PS (L1C1), anti-*D. agitata* CKB (L6C1) and anti-*D. aromatica* RCB (L11C1) were type A.II.b antibodies: they were not in the sample, and their spot brightness was due to a nearby microorganism or molecule that is certainly not represented in the graph. Conversely, anti-*M. bellicus* VDY (L2C1), anti-*I. dechloratans* (L3C1), anti-*D. hydrogenophilus* LT-1 (L4C1), anti-*Propionivibrio militaris* MP (L5C1), anti-*Magnetospirillum* sp. WD (L7C1), anti-*S. algae* (L9C1), anti-*D. chlorophilus* NSS (L10C1) and *Arcobacter* sp. CAB (L12C1) were type A-III or B-III antibodies, and therefore the corresponding target microorganisms or molecules (or at least other taxonomically close related ones) were actually in the sample.

In order to present the potentiality of the deconvolution method, let us analyze some cases of particular importance. The deconvolution yields that neither anti-*A. suillum* PS (L1C1) or anti-*Azospira* sp. ZAP (L8C1) were in the sample, because in that case they would cross-react with anti-*A. suillum* PS EPS (L1S2), as shown in the antibody graph plotted in Figure 4B, but the latter showed no fluorescence (Figure 5A). In a similar way, we obtained that anti-*D. aromatica* RCB (L11C1) was not in the sample because it would cross-react with anti-*D. aromatica* RCB EPS (L11S2), which again showed no fluorescence. Finally, let us note that, despite its large signal, anti-*D. agitata* CKB (L6C1) was not in the sample, as it would cross-react with anti-*D. agitata* CKB EPS (L6S2) more intensively than it does. The positive fluorescence of L6C1 was therefore due to the existence of a close relative.

DNA extractions were performed for 16S rRNA gene sequencing and analysis from different American Pacific^TM^ reactors samples (Supplementary Table 2). Up to 10 operational taxonomic units (OTU) identified were attributed to potential dissimilatory PRB of the *Betaproteobacteria* class, and particularly of the *Rhodocyclales* order, the same order to which *D. agitata* CKB belongs to. This fact is in full agreement with the results obtained from the deconvolution method described above. Therefore, we assume that the biomass of some related native strains including the produced EPS material, which are not present in the PRBCHIP, could be targets for the L6C1 and L6S2 antibodies.

Similarly, we tested the ability of PRBCHIP for detecting potential PRB in other environments. To test possible cross-reactions with minerals present in soil material, we used negative controls consisting of subjecting samples to a heat treatment to destroy the organic matter potentially present. The samples were analyzed in parallel with the non-treated ones and the final fluorescence intensities were calculated by subtracting the fluorescence intensities from the negative controls to the non-treated samples. Fluorescent signals either completely disappeared or were substantially reduced after heat treatment, which indicates that fluorescence was indeed due to organic molecules present in the samples. Samples from the salt precipitates around the Lost Hammer perennial springs were tested with PRBCHIP in a FSMI and revealed with the TOP-PRB antibody mixture. The results showed weak immunodetection signals for most of the antibodies for LH-Sed sample (Figure 5B), being anti-*D. agitata* CKB (L6C1) and anti-*Arcobacter* sp. CAB EPS (L12S2) the antibodies with the largest fluorescence intensity values.

The deconvolution method applied to sediment sample LH-Sed (Figure 5B and section “Materials and Methods”) indicated that anti-*I. dechloratans* (L3C1), anti-*Azospira* sp. ZAP (L8C1) and anti-*Arcobacter* sp. CAB EPS (L12S2) were type A.II.a or B.II antibodies, so their cognate antigens were not in the sample, and their brightness was due to a nearby microorganism or molecule that can be represented in the graph or not; anti-*Arcobacter* sp. CAB (L12C1) was a type A.II.b antibody, which means that it was not in the sample, and its brightness was due to a nearby microorganism that is not represented in the graph; finally, anti-*A. suillum* PS (L1C1), anti-*A. suillum* PS EPS (L1S2), anti-*M. bellicus* VDY (L2C1), anti-*D. hydrogenophilus* LT-1 (L4C1), anti-*P. militaris* MP (L5C1), anti-*D. agitata* CKB (L6C1), anti-*D. agitata* CKB EPS (L6S2), anti-*Magnetospirillum* sp. WD (L7C1), anti-*S. algae* (L9C1), anti-*D. chlorophilus* NSS (L10C1), anti-*D. aromatica* RCB (L11C1) and anti-*D. aromatica* RCB EPS (L11S2) were type A.III or B.III antibodies, that is, they were actually in the sample, or a nearby organism was.

It is remarkable that the fluorescence associated to antibodies anti-*I. dechloratans* (L3C1) and anti-*Azospira* sp. ZAP (L8C1) were due to cross-reactions with anti-*S. algae* (L9C1) and anti-*A. suillum* PS (L1C1), respectively (see the antibody graph, Figure 4B). Therefore, it is highly probable that the species used as immunogen to produce anti-*A. suillum* PS (L1C1), or a very close relative, and that of anti-*S. algae* (L9C1), were indeed present in the sample, while *Azospira* sp. ZAP or *I. dechloratans* strains were not.

**FIGURE 6:**
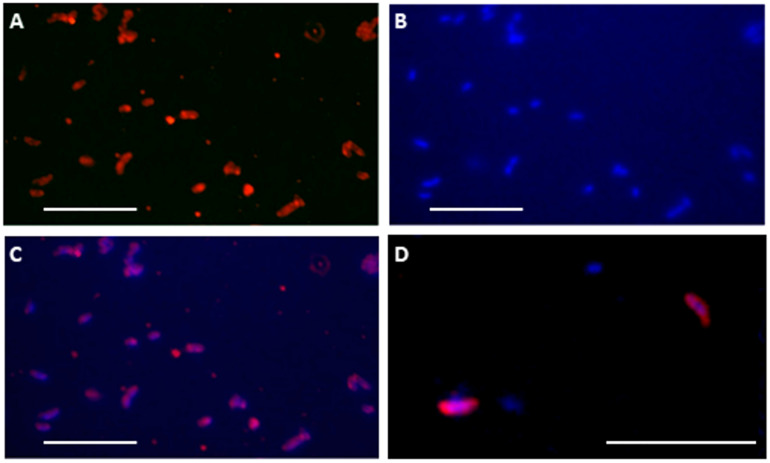
A-Cld antibody binds and detects periplasmic Cld protein in culture enrichments and natural samples. Fluorescent cell immunostaining with anti-Cld antibody **(A)** and DAPI staining **(B)** of cells obtained after a 4°C culture enrichment of a natural sample from the High Arctic sample (LH-SC). Overlapped images **(C)** showed the correlation of immunostaining with nucleic acids in comma-like cellular morphologies. **(D)** Merged A-Cld antibody and DAPI stains showing cells extracted directly from the High Arctic LH-SC sample. White bar: 5 microns.

Besides the positive immunodetection with anti-crude cellular extracts, positive immunodetection was also observed in the four antibodies to perchlorate reductase and Cld proteins in other LH samples (not shown), indicating that these protein fragments or cells producing them were present in the sample. Although none of the 16S rRNA gene sequences were attributed to the strains on the PRBCHIP, some DNA sequences retrieved with specific amplification of the *cld* (Watanabe et al., 2001) gene from LH samples revealed the presence of *D. agitata cld*-like genes (Supplementary Table 1), in agreement with the PRBCHIP results and the deconvolution method that confirmed the presence of this microorganism or a closely related one (or with the same enzymatic activity) in the sample (L6C1 and L6S2 in Figure 5B). Moreover, cells from LH-SC sample were fluorescently stained (Figure 6D; see section “Materials and Methods”) with anti-Cld antibody. Similarly, cells from a 4-months culture enrichment under anaerobic conditions in perchlorate medium at 4°C also positively stained with the fluorescent anti-Cld antibody (Figure 6). DNA extraction from the culture enrichment and 16S rRNA gene sequencing revealed the predominance of *Gammaproteobacteria* with less than 93% identity to the closest known strains such as *Thioalbus, Salinispirillum, or Thioalkalivibrio* against the reference sequence (RefSeq) database from the “Basic Local Alignment Search Tool” (BLAST; GeneBank accession Numbers MN228659-MN228691). Although there were no antibodies printed in the FSMI associated to these three genera, the deconvolution method yielded the existence of the *Gammaproteobacteria Shewanella algae* (L9C1 in Figure 5B, or a relative) which would be compatible with these results.

## Discussion

### A Multiplex Immunoassay for Detecting Perchlorate-Reducing Bacteria (PRB)

Perchlorate ions are important pollutants on Earth and the understanding of how microbes remove them from anaerobic environments is of general interest. Our PRBCHIP immunosensor could be an appropriate tool for detecting the type of microorganisms associated with perchlorate removal through specific immune-identification in natural samples with high sensitivity and relatively high taxonomic resolution (Table 1). The PRBCHIP LOD ranged from 500 to 10^6^ cells mL^–1^, depending on the performance of each antibody, being for most of them between 10^3^ to 10^4^ cells mL^–1^. Some cross-reactivity was detected between antibodies raised against strains and EPS from the same genus, as it happened between the anti-*Azospira* species, or between anti-*Dechloromonas* spp. and anti-*Arcobacter* sp. CAB. Minor and weak cross-reactions were observed between anti-*Magnetospirillum* sp., anti-*S. algae*, and anti-*D. chlorophilus* antibodies and their immunogens (Figure 4). The lower detection limits (10^2^–10^3^ cells mL^–1^) suggest high affinity and/or specificity of the antibodies against their cognate antigen, and probable more diverse antigen sites present per cell, making PRBCHIP a very sensitive tool for monitoring perchlorate-reducing bacteria.

The PRBCHIP cross-reactivity test, together with the antibody graph and the associated deconvolution analysis, indicated that, in general, this antibody collection is specific and has certain taxonomical value, in spite of the fact that the antibodies were produced using whole cell lysates or crude EPS fraction as immunogens. The probability of different species, even genera, of sharing some cellular or extracellular immunogenic component is relatively high and, consequently, the antibodies showed certain level of interaction even with apparently unrelated strains.

Because the immunogens used consisted of complex crude lysates or EPS preparations, we cannot rule out the possibility that the antibodies are recognizing proteins involved in several functions as may be proteins involved in perchlorate respiration. Although these proteins have small differences between microorganisms, they indeed keep conserved domains that could be the reason of the cross-reactivity between the antibodies and different strains in the PRBCHIP assays (Melnyk et al., 2011, Melnyk and Coates, 2015). The analysis of the antibody graph and its associated deconvolution method disentangle such existing cross-reactivities, and therefore help to identify the true cognate immunogen for each antibody.

No positive results with the antibodies against PcrAB and Cld proteins (A-PCR A/B, A-Cld, A-295, and A-PCR) were obtained by FSMI, although a dot-blot assay indicated that the immunodetection worked appropriately (Figure 3). A possible explanation of this behavior is that these proteins can be captured by the immobilized capturing antibodies on the PRBCHIP, but might not exhibit any free site (epitopes) for the tracer antibody binding. However, these antibodies specifically bound to some component from *D. agitata* CKB and *D. alkaliphilus strains*. It is probable that the epitopes (part of the proteins that are recognized by the antibody) of these proteins are more exposed as part of the crude cell extracts where the proteins can be partially unfolded or denatured.

In a direct dot-blot assay, however, the epitopes are exposed and available for binding to the tracing antibodies. In fact, whole-cell extracts from *D. agitata* and *D. alkaliphilus* contained specific immunoreactive material with the anti-protein antibodies, as they showed positive immunodetection by FSMI (Table 2), indicating that larger protein complexes, oligomeric forms, or membrane complexes bearing these proteins are indeed exhibiting accessible epitopes of these proteins. In fact, immunostaining of whole cells with anti-Cld antibody (Figure 6) confirmed this explanation: whole cells act as a multi-epitope containing complex so that immobilized antibodies on the PRBCHIP capture the cells and still there are multiple free epitopes for forming the sandwich with the fluorescent antibody.

The PRBCHIP was validated with an extensive strain collection, which included chlorate and perchlorate reducers, or sulfur reducers (Table 2), grown in aerobic and anaerobic conditions. The results indicated that most antibodies were strains of genera specific, and they can detect the presence of proteins involved in the perchlorate reduction, which only appear when the microorganisms are cultivated under anaerobiosis (Table 2). The efficiency and specificity of the PRBCHIP were demonstrated using complex mixtures of perchlorate-reducing bacteria by FSMI, by distinguishing the corresponding strain even when we used the TOP-PRB antibody tracer mixture.

The PRBCHIP was also efficient in testing natural samples by specifically detecting the presence of *D. agitata* CKB in perchlorate contaminated samples, even when the DNA OTU analysis only distinguished at the genus level. Not only were we able to detect potential reducing perchlorate activity in the samples, but also to identify the putative microorganisms involved on it. Also, we detected perchlorate reducing strains related with *D. agitata* in perennial springs at Canadian High Arctic, although no perchlorate was detected by ion chromatography (not shown). We cannot infer whether these bacteria are indeed reducing the potential minimal amounts of perchlorate, however, PRBCHIP results showed an unequivocal sign of the presence of these bacteria or other related strains. The retrieval of *cld* gene sequences as well as the immunostaining of cells from LH-SC sample with anti-Cld antibody confirmed its presence in the salt precipitates. The role of the Cld protein might be more associated with detoxifying activities from Cl^–^ ions than dissimilatory perchlorate reduction (Hatzinger, 2005; Aziz and Hatzinger, 2009; Wang and Coates, 2017).

Additionally, the antibody graph and the deconvolution method helped us to disentangle the PRBCHIP signals and obtain the ones corresponding to the cognate immunogens (Figures 4, 5) (Rivas et al., 2011; Blanco et al., 2015b); in spite of being polyclonal antibodies they exhibited a relatively high specificity. The production of new antibodies to new strains or specific components will strength the PRBCHIP phylogenetic character and will increase its value for environmental monitoring. This is especially relevant because the ability to use perchlorate as electron acceptor is widespread among bacteria belonging to a broad phylogenetic affiliation. Further work is necessary to find common traits or compounds in order to develop generic probes for detecting perchlorate reducing microorganisms regardless of their phylogenetic affiliation.

### Perchlorate-Reducing Bacteria and Implication for Life Detection on Mars

Perchlorate-reducing bacteria have been studied for years due to their relevance in removing the perchlorate pollution from the environment (Wang and Coates, 2017). The discovery of the hyperthermophilic archaea *Archaeoglobus fulgidus*, capable of reducing perchlorate and chlorate, in an underwater volcanic vent that might be close to the conditions on Earth more than 2.5 billion years ago (Liebensteiner et al., 2013), suggests that perchlorate and chlorate could have been present in the early history of Earth. It has been proposed that perchlorate reduction may have developed very early in the evolution of life, when the Earth’s atmosphere had no oxygen, being thus widespread in the prokaryotic world (Liebensteiner et al., 2013). In this period, perchlorate reduction formed chlorite in the cytoplasm, and because of its toxicity had to be reduced into chloride (Cl^–^) and oxygen (O_2_). Youngblut et al. (2016) proposed that primary Cld function probably evolved independently as a means of detoxifying naturally occurring chlorine produced either abiotically or biologically by non-specific ancestral reductases. Therefore, the oxidative stress associated with the reduction of perchlorates as well as the generation of reactive carbonyl species (RCS) could lead to the specialization of Cld. Phylogenetic analyses based on 16S rRNA and the primary functional genes involved in perchlorate metabolism (Cld and PCR) together with the evolutionary history of a perchlorate reduction genomic island (PRI) (Hofbauer et al., 2014; Melnyk and Coates, 2015) support the idea that both enzymes evolved once in a short period, and probably, through horizontal gene transfer (HGT), contributed to expanding this metabolic activity among diverse microbial groups (Bender et al., 2004; Bardiya and Bae, 2011).

Since the discovery of a relatively high concentration of perchlorate on the surface of Mars (Hecht et al., 2009), even possibly present in deep (1.5 km depth) brines (Orosei et al., 2018; Lauro et al., 2020), PRB have gained special relevance as potential inhabitants of the perchlorate-rich Martian near shallow or deep subsurface. Perchlorate (ClO_4_^–^) could have been used as electron acceptor for hypothetical martian PRB, while other microbial species just acquired the detoxifying capabilities of enzymes such as the Cld to protect themselves from the poisonous effects of other species of chlorine (ClO_3_^–^, ClO_2_^–^) (Rikken et al., 1996; Mlynek et al., 2011; Hofbauer et al., 2014).

Besides the use of PRBCHIP as an immunosensor for the detection of PRB in contaminated or natural terrestrial environments, it can be easily implemented into instruments for *in situ* life detection in planetary exploration, such as SOLID (Signs of Life Detector) (Parro et al., 2005, 2011a,b; Rivas et al., 2008). SOLID automatically processes 0.5 g of soil or powder and extracts the organic and biological material into a liquid solution/suspension by ultrasonication. After a filtering step to remove the coarse material, the extract is directly incubated with the LDChip (Life Detector Chip), an antibody microarray that can contain more than 200 antibodies raised against microbial biomarkers relevant for planetary exploration (Rivas et al., 2008; Parro et al., 2011b; Blanco et al., 2013, 2017). SOLID is a high TRL (Technological Readiness Level) instrument highlighted by the NASA’s Mars 2020 science definition team as strawman instrument for biomarker detection in planetary exploration (McKay et al., 2013). Antibodies to PRB and their characteristic protein markers are part of LDChip and might be useful for the search for molecular evidences of a mature, evolved, prebiotic chemistry or biochemical evidences of life on mission concepts as the proposed IceBreaker mission to the Martian northern permafrost (Mustard et al., 2013).

### Specificity and Shot-Gun Approach of the Bio-Affinity Techniques for Life Detection

Besides the metabolic-based experiments on the Viking landers, the detection of signs of life has been limited to the search for organic molecules by analyzing volatile compounds by gas chromatography-mass spectrometry (GC-MS) in different missions to Mars (Klein et al., 1976; Biemann et al., 1977; Mahaffy et al., 2012). So far, only the SAM instrument on board of rover Curiosity has detected organic compounds in the form of derivatives of benzene, thiophene and several aliphatic hydrocarbons (Eigenbrode et al., 2018). NASA’s Mars 2020 and ESA’s ExoMars rover also include spectrometric analytical techniques such as Raman (Rull et al., 2017), and Infrared, and laser desorption coupled to MS in the case MOMA instrument (Goesmann et al., 2017) for ExoMars. Major advantages of these techniques reside in easy sample or no sample processing, the capability for multiple analysis, and their general, not predetermined agnostic detection of molecules. However, in general, the size and the complexity of the detected molecules is relatively small (<1,000 Da), being their diagnostic capability, and the ability to inform about their taphonomic history, very limited.

Bio-affinity based techniques such as antibodies complement those techniques. Antibodies can be highly specific, capable of discriminating chiral compounds (Hosftetter et al., 1999; Kassa et al., 2011; Moreno-Paz et al., 2018) or to recognize universal structures such as DNA (Hu et al., 2014). We rely on these properties, the polyclonal nature of antibodies, and the capability of multiplexing the immunoassay, to implement both PRBCHIP and LDChip in a *shot-gun* strategy that increases the probability to detect simple and complex biomolecules. We produce polyclonal antibodies to recognize from the most universal molecules of life (e.g., DNA, peptidoglycan) to other more specific such as evolutionary well-conserved proteins from the most ancient metabolic pathways. Every single spot on the chip contains thousands of antibody molecules able to bind a biological polymer or a microbial cell through different chemical structures, some of them unique, some shared with others. Such a broad specificity is not appropriate for taxonomic proposes, but it increases the probability to detect related compounds or strains. This, together with the multiplexity, that allows redundancy in the number and type of antibodies, and the antibody graph with its deconvolution method associated, make PBRCHIP and LDChip powerful tools for complementing the more generalist chromatographic and spectroscopic techniques in planetary exploration. LDChip is a sort of small “immune system” trained for targeting thousands of molecular structures found in terrestrial analog environments where there are bacteria dealing with perchlorate metabolism.

## Conclusion

Herein, we have shown a new and highly sensitive antibody microarray, the PRBCHIP, which can be useful for multiplex detection and identification of perchlorate reducing bacteria with relative taxonomic resolution. PRBCHIP may be effective for in-field detection and monitoring PRB in natural and contaminated terrestrial ecosystems and could be a valuable technique for large monitoring programs for acquiring inexpensive and robust datasets. In addition, the collection of antibodies can be implemented to be used for *in situ* life detection in planetary exploration, particularly Mars, where relatively high perchlorate concentration has been detected and might be used by potential Martian bacteria for dissimilatory perchlorate reduction. Current PRBCHIP is just the core of an extending and adaptive system that can be improved as a function of the potential application.

## Data Availability Statement

The datasets generated for this study can be found in online repositories. The names of the repository/repositories and accession number(s) can be found below: https://www.ncbi.nlm.nih.gov/genbank/, MN228659–MN228691.

## Author Contributions

MM-P, VP, and LR conceived and designed the project. AE, EA-P, IG-C, LR, MM-P, and YB performed the experiments. IG-C, JA, MM-P, VP, and YB analyzed the data. AE, JA, CM, and IR-B contributed with reagents, materials, and analysis tools. IG-C, JA, MM-P, and VP wrote the manuscript. LW contributed to improving the manuscript. All authors contributed to the discussion and interpretation of the results.

## Conflict of Interest

LA was employed by company Inmunología y Genética Aplicada, S.A. (INGENASA). The remaining authors declare that the research was conducted in the absence of any commercial or financial relationships that could be construed as a potential conflict of interest.
