# Supplementary material for: A Multiplex Immunosensor for Detecting Perchlorate-Reducing Bacteria for Environmental Monitoring and Planetary Exploration
Source: Front Microbiol. 2020 Dec 16;11:590736. doi: 10.3389/fmicb.2020.590736 (PMC7772991; doi:10.3389/fmicb.2020.590736)
Supplement: Supplementary file 1 [file Data_Sheet_1.docx]

**SUPPORTING INFORMATION**

**A multiplex immunosensor for detecting perchlorate-reducing bacteria for environmental monitoring and planetary exploration.**

Ignacio Gallardo-Carreño^$^, Mercedes Moreno-Paz^$^, Jacobo Aguirre, Yolanda Blanco, Eduardo Alonso-Pintado, Isabelle Raymond-Bouchard, Catherine Maggiori, Luis A. Rivas, Anna Engelbrektson, Alfonso F. Davila, Lyle Whyte, and Víctor Parro.

This section contains 2 tables.

| **Ab name** | **OD** **600 nm** | **Cells/mL** | **Description** | **16 S rRNA gene Accession N^o^** |
| --- | --- | --- | --- | --- |
| L1C1 | 0,166 | 1,33E+08 | *Azospira* suillum PS | NR_074103.1 |
| L2C1 | 0,1388 | 1,07E+08 | *Magnetospirillum bellicus* VDY | NR_116009.1 |
| L3C1 | 0,1857 | 1,49E+08 | Ideonella sp. EP3-2L | KT619177.1 |
| L4C1 | 0,2357 | 1,89E+08 | *Dechlorobacter hydrogenophilus* LT-1 | NR_125499.1 |
| L5C1 | 0,1992 | 1,59E+08 | *Propionibrio*  *militaris* MP | NR_125528.1 |
| L6C1 | 0,1734 | 1,39E+08 | *Dechloromonas agitata* CKB | AF170354. |
| L7C1 | 0,1388 | 1,07E+08 | *Magnetospirillum sp.* WD | AAT07043.1 |
| L8C1 | 0,2914 | 2,34E+09 | *Azospira sp.* ZAP | 2508501008 |
| L9C1 | 0,1746 | 1,40E+08 | *Shewanella algae* LTY2 | KC210854.1 |
| L10C1 | 0,3922 | 3,14E+08 | *Dechloromarinus chlorophilus* NSS | KP122946.1 |
| L11C1 | 0,1487 | 1,19E+08 | *Dechloromonas aromatica RCB* | CP000089.1 |
| L12C1 | 0,317 | 2,55E+09 | *Arcobacter sp. CAB* | KP137431.1 |

**Table S1**. Perchlorate reducing bacterial strains used as immunogens as identified by their 16S rRNA gene sequence.

**Table S2.** Percentage of different OTUs identified from 16S rRNA gene sequences in different American Pacific^TM^ reactors from contaminated soils (A-N). See text for explanation.

| **OTU nº** | **Order*** | **Family** | **Genus** | **A** | **B** | **C** | **D** | **E** | **F** | **G** | **H** | **I** | **J** | **K** | **L** | **M** | **N** |
| --- | --- | --- | --- | --- | --- | --- | --- | --- | --- | --- | --- | --- | --- | --- | --- | --- | --- |
| 2 | Burkholderiales | Comamonadaceae | *Variovorax* | 3,3 | 0,5 | 2,1 | 0,5 | 0,4 | 2,0 | 6,4 | 2,3 | 7,0 | 3,3 | 0,6 | 2,6 | 0,3 | 0,1 |
| 6 | Burkholderiales | unclassified | unclassified | 9,9 | 0,6 | 10,4 | 0,3 | 0,5 | 9,5 | 4,4 | 2,5 | 5,8 | 6,4 | 0,8 | 2,8 | 4,7 | 1,8 |
| 19 | Rhodocyclales | Rhodocyclaceae | *Ferribacterium* | 1,2 | 0,4 | 0,8 | 0,4 | 0,4 | 1,0 | 0,6 | 1,0 | 0,2 | 2,8 | 0,3 | 0,7 | 1,3 | 0,1 |
| 20 | unclassified | unclassified | unclassified | 0,0 | 0,0 | 0,0 | 0,0 | 0,0 | 0,0 | 0,1 | 0,0 | 0,0 | 1,1 | 0,0 | 0,0 | 1,0 | 0,1 |
| 25 | Rhodocyclales | Rhodocyclaceae | unclassified | 0,9 | 0,9 | 0,5 | 1,4 | 1,5 | 0,6 | 1,9 | 1,7 | 1,0 | 1,4 | 4,0 | 0,7 | 2,9 | 1,3 |
| 27 | Burkholderiales | unclassified | unclassified | 2,6 | 0,0 | 0,0 | 0,9 | 1,0 | 2,0 | 3,5 | 0,6 | 0,0 | 0,0 | 0,0 | 0,0 | 0,6 | 0,0 |
| 29 | Burkholderiales | Comamonadaceae | *Hydrogenophaga* | 2,5 | 0,8 | 1,9 | 0,5 | 0,6 | 1,5 | 0,9 | 1,3 | 1,0 | 3,1 | 0,6 | 1,2 | 0,0 | 0,0 |
| 30 | Rhodocyclales | Rhodocyclaceae | *Zoogloea* | 1,9 | 0,4 | 1,6 | 0,6 | 0,7 | 2,1 | 1,0 | 0,4 | 0,6 | 1,0 | 1,3 | 0,4 | 0,8 | 0,0 |
| 41 | unclassified | unclassified | unclassified | 0,0 | 2,5 | 1,4 | 0,0 | 0,0 | 0,0 | 0,0 | 0,0 | 2,8 | 4,6 | 0,9 | 0,3 | 0,0 | 0,2 |
| 89 | unclassified | unclassified | unclassified | 0,0 | 0,0 | 0,0 | 0,0 | 0,0 | 0,0 | 0,0 | 0,0 | 0,0 | 0,0 | 0,0 | 0,0 | 1,1 | 2,2 |

All identified OTUs are Betaproteobacteria, some of them from unclassified order.
